# Possible Emergence of Sequence Specific RNA Aminoacylation via Peptide Intermediary to Initiate Darwinian Evolution and Code through Origin of Life

**DOI:** 10.3390/life8040044

**Published:** 2018-10-02

**Authors:** Dimiter Kunnev, Anastas Gospodinov

**Affiliations:** 1 Roswell Park Cancer Institute, Department of Molecular & Cellular Biology, Buffalo, NY 14263, USA; dimiter.kunnev@roswellpark.org; 2 Roumen Tsanev Institute of Molecular Biology, Bulgarian Academy of Sciences, Acad. G. Bonchev Str. 21, Sofia 1113, Bulgaria

**Keywords:** origin of life, hybridization-dependent peptide, origin of genetic code, translation, ribozymes, RNA world, RNA-peptide world

## Abstract

One of the most intriguing questions in biological science is how life originated on Earth. A large number of hypotheses have been proposed to explain it, each putting an emphasis on different events leading to functional translation and self-sustained system. Here, we propose a set of interactions that could have taken place in the prebiotic environment. According to our hypothesis, hybridization-induced proximity of short aminoacylated RNAs led to the synthesis of peptides of random sequence. We postulate that among these emerged a type of peptide(s) capable of stimulating the interaction between specific RNAs and specific amino acids, which we call “bridge peptide” (BP). We conclude that translation should have emerged at the same time when the standard genetic code begun to evolve due to the stabilizing effect on RNA-peptide complexes with the help of BPs. Ribosomes, ribozymes, and the enzyme-directed RNA replication could co-evolve within the same period, as logical outcome of RNA-peptide world without the need of RNA only self-sustained step.

## 1. Introduction

Life on Earth appeared 3.7–4.1 b. y. ago, shortly after the planet formed [1,2,3,4]. Any plausible hypothesis for the origin of life should explain the formation of a system capable of Darwinian evolution. The accepted sequence of events leading to formation of such a system could be summarized as follows: (1) Increased chemical complexity led to RNA or RNA-like oligomers; (2) together with the prebiotic amino acids, RNAs gave rise to the proto-ribosome, metabolism and membrane separation; (3) these led to the formation of the Last Universal Common Ancestor (LUCA) as a primordial ancestor of all life on Earth today.

Many hypotheses adopt a specific process—such as RNA replication, metabolism or membrane formation—as the organizing force of early events leading to the origin of life [5]. An overarching need is to establish interactions between the carrier of genetic information and that of structure/function. It is unclear how the initial relationships between RNA and proteins came to being, bearing in mind that RNA needs proteins to replicate and proteins need RNA to be coded (a problem known as “chicken and egg dilemma”).

An attempt to explain this dilemma is the RNA world hypothesis [6,7,8,9,10,11], in which RNA assumes both catalytic (ribozyme) and hereditary information storage functions [6,12,13] and it is thus both self-sustained and capable of Darwinian selection. RNA world hypothesis is widely accepted today, having various versions with the common assumption that the existence of RNA only sustained system predates RNA/protein and RNA/DNA/protein systems [6,7,8,9,10,11]. In strong support of the RNA world are the many ribozymes selected under laboratory conditions for almost all the steps of translation (reviewed in Reference [9]) as well as for RNA dependent RNA polymerization [14,15,16,17,18,19,20]. In addition, the ribosome itself and many RNA relics are capable of catalytic functions on their own (natural ribozymes), without proteins [12,13,21]. The peptidyl transfer center (PTC) of the large ribosome subunit (LSU) is free of proteins to a distance of 17 Å of the catalytic site [22,23] (reviewed by Harry Noller [24]) and most of the extant ribosomal proteins are peripheral with heterogeneous origin.

Another argument used to support the RNA world hypothesis is the existence of different aminoacyl-tRNA synthetases (aaRS) for the same tRNA (Cys) found in methanogenic archaea and some bacteria [25,26]. According to Söll and coworkers, this indicates that the two different classes of aaRS have adapted independently to the tRNA(Cys) and that the evolution of the genetic code and the aaRSs were initially unrelated [26,27]. This hypothesis also states that at first aminoacylation was performed by ribozymes, which were later supplanted by aaRS. It is not clear, however, what kind of selection pressure could have led to the transition to a totally different tRNA-amino acid recognition system.

Despite the general agreement among supporters of RNA world hypothesis that a self-sustained ribozyme should have existed independently before the RNA/protein world came into being, there are a number of caveats with the hypothesis pointed so far [5,10,28,29,30]. The RNA world hypothesis also does not offer a commonly accepted path to the first self-sustained RNA world system capable of Darwinian selection. There are various hypotheses about the pressure that drove the selection of RNAs capable of ribozyme replication [6,7,8,9,10,11]. There are also various mechanisms suggested of how RNA-directed polymerization (the “Holy Grail” of RNA world) occurred: (1) By RNA polymerase ribozyme and [31,32,33,34,35,36]; (2) by the addition of triplets from hybridized tRNA-like molecules to the growing chain of RNA as Penny and Poole suggested [7]; or (3) the proto-ribosome started as RNA replication ribozyme that mediated ligation of trinucleotides in response to an RNA template [24,37].

Irrespective of whether an RNA-only self-sustained system capable of Darwinian selection is possible, the key question in the origin of life as we know it is how translation of genetic information came into being? Several mechanisms establishing correspondence between codons and their cognate amino acids have been suggested, either direct or via RNA adaptors.

Most of these are based on stereochemical compatibility (“code”) [9,38,39,40,41,42,43,44]. As an example, the Direct RNA Template (DRT) hypothesis proposes interactions of adjacent stereochemically compatible amino acids along the RNA sequence [43]. Ma suggested the interaction of template RNA and amino acids via RNA adaptors to lower replication costs of the DRT model [44]. Another hypothesis suggests a triplet RNA replicase/polymerase as a precursor of the ribosome [7]. These theories that attempt to explain the emergence of translation are summarized in an excellent review by Koonin and Novozhilov [45]. While these hypotheses offer possible explanations of the path towards translation, they fail to answer why this mechanism is not evolutionary preserved?

On the topic of ribosome formation, advocates of the RNA world hypothesis are divided as well: Some believe the peptidyl transferase center first appeared in the large ribosomal subunit (LSU) [9,10,24,37,46], while others consider the small one (SSU) as the first codon recognition site [7]. The peptidyl transferase center (PTC) as part of LSU is considered a derivative of a ribozyme capable of RNA replication [37] or peptidyl transfer [9,10,46]. Another hypothesis places proto-tRNAs at the center of a positive feedback loop in which these via concatenation and modification gave rise to both early genes and the PTC. The peptides produced by proto-translation stabilized the proto-RNA complex, leading to selection and diversification of proto-tRNA into mRNA and rRNA [47].

All hypotheses for the origin of life incorporate peptides as stabilizing factor, the differences being in the role they played in code formation. All variants of RNA world hypothesis by definition exclude the peptides from the initial events and place the involvement of peptides after code formation, with a variety of explanations of polymer transition and ribosome formation. In contrast, the hypotheses that reject the RNA world postulate that the peptides should have been part of the very first steps in the establishment of the genetic code [28,48,49,50].

The two sets of theories also generally differ on the process that initiated Darwinian selection. The non-RNA world hypotheses theories put forward the emergence of proto-translation system, while the RNA world ones assume RNA replication as the trigger of selection. However, the gene set of the LUCA is thought to include largely highly conservative genes involved in translation [51,52], giving us an important clue that the translation complex could be the very first subject of Darwinian evolution.

Recently, Williams and colleagues rejected the RNA world and proposed a hypothesis in which the PTC in combination with peptides was central to the origin of life. The PTC originated via stabilizing interactions between peptides and RNA and subsequently evolved into the ribosome [28]. In their scenario, the proto-ribosome predated RNA replication making the RNA world not necessary. In response, Poole and co-authors argued that while non-coded peptides certainly played a role in early stages of evolution, it is unlikely that the PTC center could have evolved in the absence of Darwinian evolution utilizing heredity. According to Poole and Penny the heredity may come only from an RNA world scenario [53]. It thus seems that the emergence of specific aminoacylation is the major problem, which needs to be solved since this process is the foundation of any bio-heredity as we know it.

In the post-LUCA time, there is only one type of system capable to recognize and couple specific amino acids to specific tRNAs: Amino-acyl transferases (aaRS class I and aaRS class II). The genetic code is embedded into the aaRS structure putting these proteins in the unique position as the only ones to “know” the genetic code. Even so, aaRS are mostly dismissed from the initial processes of codon formation due to variety of reasons, some of which were described above. In addition, it was shown that aaRS class I share protein HUP class domain with other proteins such as USPA, ETFP, photolyase, and PP-ATPase [54,55]. Phylogenic analysis demonstrates common origin for 15–18 distinct α/β ATPases and nucleotide-binding proteins of the HUP class. The split of HUP class domains should occur before LUCA, while all members of class I aaRS may have emerged at stage later then HUP. Koonin and team concluded that such a level of evolutionary diversity should occur before protein-dependent translation machinery, therefore into the RNA world [54].

Contrary to this view, Schimmel was the first to propose that aaRSs and the genetic code must have coevolved “getting fitted” to each other [56,57]. Recently, Charles Carter and colleagues corroborated this hypothesis suggesting that aaRSs and especially their ATP binding domain were involved in the initial events that led to the development of the genetic code [29,30,50,58,59].

We do not see controversy in the fact that the aaRS class I could evolve at a later stage before LUCA time and to share HUP class domain with another nucleotide-binding proteins. Additionally, based on the fact that today aaRSs are the only enzymes capable to aminoacylate the correct tRNAs with the correct amino acids we reason that some proto-forms of aaRSs might have played a role in codon formation. We postulate that the evolution of aaRS begun as a very short peptide that facilitated amino-acylation of RNA with low (but already not random) specificity—we call it “bridge peptide” or BP. The BP was synthesized initially by chance as a result of the physical proximity of hybridized short random amino-acylated RNAs. The bridge peptide facilitated the emergence of a self-sustained RNA-peptide complex supporting a primitive translation. This concept offers an explanation for the development of the genetic code, the ribosome, the RNA relics (natural ribozymes), and RNA replication with minimal assumptions.

## 2. Steps of the Proposed RNA-Peptide World Scenario

### Steps towards Translation

Here, we propose a hypothesis that could have led to the emergence of RNA-encoded peptide synthesis. The events described may not have been strictly sequential.

### 2.1. Assumptions for Primordial Earth Conditions

Prebiotic synthesis: As a result of chemical prebiotic expansion activated ribonucleotides [60,61], (reviewed by References [62,63]) and amino-acids [64], (reviewed by References [65,66,67]) would emerge in the early Earth conditions (reviewed by Reference [68]), [4,69]. RNA oligomer synthesis: Ribonucleotides would polymerize in acidic to neutral conditions leading to very short RNAs from 2 to ~40 bases [68,70,71,72,73]. The polymerization is not a templated process and RNAs would have random sequence and random 3D structures due to hydrogen bonding at lower temperature. Non-enzymatic template-directed RNA polymerization: We assume that it might have occurred with activated nucleotides. The process would have low fidelity and would result in the amplification of short sequences, preserving mostly stable ones. This non-enzymatic RNA polymerization (RNA replication) would be added to the stability equation without changing the core of the model [74,75]. Geological cycles: Life cannot start in steady-state conditions and would need selection of different forms. The natural thermal cycles from 0 °C or below to ~100 °C would be present and would melt and re-anneal RNA based on Watson-Crick base pairing interactions. Wet-Dry cycles could facilitate the process of RNA polymerization [68,71,72,73,76,77,78]. Compartmentalization is another important factor, since most of the described events are unlikely to occur in very low concentrations [79]. Some level of environmental separation would be expected, for example, micro-chambers out of porous surface of rocks or lipid vesicles or both. Surface adsorption might have facilitated RNA-RNA interactions, RNA-lipids interactions and some beneficial chemical reactions. Thus, clay surfaces have been shown to promote encapsulation of RNA into vesicles and grow by incorporating fatty acid supplied as micelles and can divide without dilution of their contents [80].

### 2.2. Step 1. Stability Selection of RNA Complexes

At temperatures between 1 °C and to denaturation (about 55 °C) temperature, short random RNA oligos would get stabilized via intra and intermolecular hybridization based on Watson-Crick base pairing, forming complexes of various 3D shape and size. Larger hybridized regions would confer greater stability and would be selected for. Highly self-complementary RNAs would be unlikely to exist, forcing intermolecular hybridization of short sequences and the emergence of complexes of several RNA oligos. The formation of RNA complexes also assumes a thermal cycle that would drive the process by sequential denaturation (~55–100 °C) and re-annealing (<55 °C) phases. Frequent repetition of the thermal cycle and stability selection would favor accumulation of complexes with higher degree of complementarity and higher GC content.

### 2.3. Step 2. Peptide Formation Due to Hybridization of Aminoacylated RNA Oligos

Non-enzymatic aminoacylation between 2′ or 3′ positions of ribose and activated amino acids could occur [81,82,83]. In addition, ribozymes capable of amino acid transfer from one RNA to another have been selected under laboratory conditions [84,85,86] and similar molecules could have participated in aminoacylation of RNAs.

**Figure 1:**
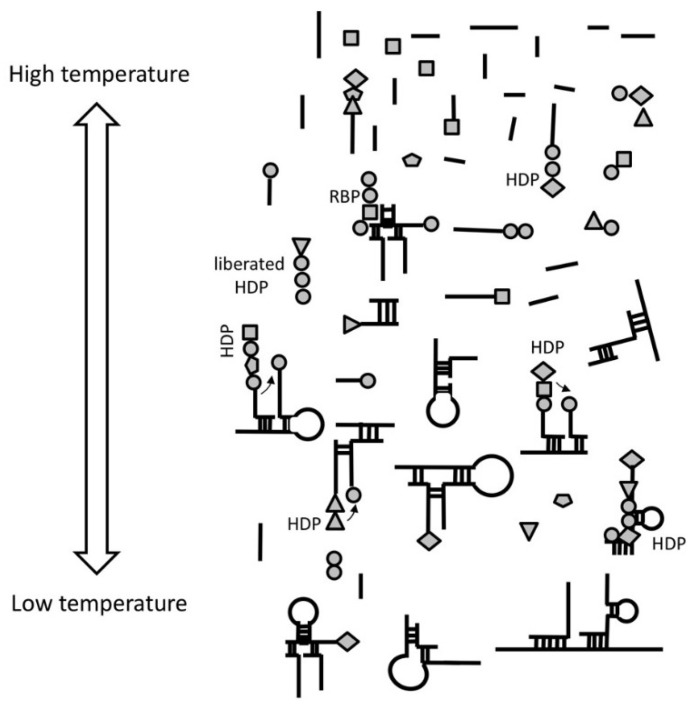
Formation of Hybridization Dependent Peptides (HDPs). The process requires thermal and wet/dry cycles to facilitate hybridization events between randomly aminoacylated RNAs. Short sequences anneal due to Watson Crick base pairing bringing RNA termini in close proximity in the newly formed complexes. This brings some amino acids (represented by circles, diamonds, triangles or pentagon shapes) close enough to form peptide bonds. During the next cycles when the temperature rises, RNA will melt and the complexes will turn into ssRNA or will degrade. Previously formed peptide will stay with RNA or will be liberated. In every cycle, a new amino acid may be added to the existing di, tri, or more amino acid-containing peptides, allowing the formation of longer HDPs (2–5 amino acids). Given the fact that that the process of aminoacylation of RNA is random, we expect newly formed HDPs to be random with compositions following amino acids abundance. Some of the HDPs would possess the ability to bind and stabilize RNA. We call these RNA-binding peptides (RBPs).

Aminoacylated RNAs would be involved in complex formation (as in step 1.), bringing some of the aminoacylated RNA 3′-ends in close proximity. This would promote peptide bond formation between two adjacent amino acids (Figure 1), most likely with the assistance of wet/dry natural cycles. All amino acids would have statistically equal probability to aminoacylate RNA. At that stage, any RNA molecule could be aminoacylated and could serve as a template. The result of the peptidyl transfer reaction between adjacent hybridized aminoacylated RNAs would be the formation of short random peptides (2 to 5 amino acids), which we call hybridization-dependent peptides (HDPs).

### 2.4. Step 3. the Amino Acid Content of HDPs 

Aminoacylation of RNAs would be governed by the availability of amino acids. Different experiments simulating prebiotic Earth conditions have proposed the molar ratios of amino acids that could have existed, reviewed in References [65,66]. While different initial prebiotic conditions might have produced different amino acids ratios, we can surmise that the most abundant amino acids would have been glycine (Gly) and Alanine (Ala) followed by Isoleucine (Ile) and Valine (Val), with the rest of amino acids present in 2 to 100 times less amounts, determining the probability for aminoacylation of RNAs. We call this probability the “chemical code” for the synthesis of HDPs. In contrast to the broadly accepted view [87,88,89] that the primordial amino acids participating in the first translation were only the first 10 most abundant ones, we propose that certain less abundant amino acids might have had a significant role as well. 

### 2.5. Step 4. Peptide-Dependent Stability

The most “useful” property of HDPs would be to increase the stability of RNAs binding them. Under primordial conditions, in the absence of specific selection, increased time of existence (as a consequence of greater stability) is a measure of fitness of the complexes. In line with this thinking, several studies have suggested that the major role of early peptides was not catalytic, but to stabilize RNA [90,91,92]. The most abundant amino acids like glycine, alanine, valine are either hydrophobic or have no charge and therefore most of the HDPs (being either hydrophobic or neutrally charged) would not stabilize significantly RNA. In contrast, incorporation of some positively charged basic amino acids such as arginine or lysine would make the HDP more capable to interact with negatively charged RNA and would increase the stability of the RNA-peptide complexes [91]. It has been shown that some peptides—selected via in vitro evolution—are significantly more efficient in stabilizing RNA structures and aiding their activities [90] and these could have been selected naturally [44].

**Figure 2:**
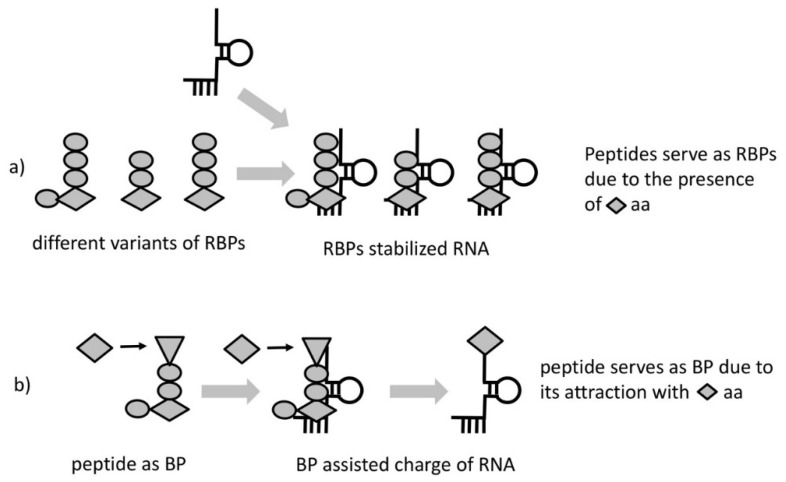
RNA binding peptides (RBPs) and bridge peptides (BP). (**a**) RBPs are HDPs which contain amino acid (represented by diamond shape) facilitating the interaction with RNA. HDPs will interact and stabilize the RNA they interact with. (**b**) A modified variant of RBP serves as BP due to its triangle aa attracting a diamond aa to RNA, facilitating de-novo aminoacylation. The function of the BP is to increase the chance of a particular amino acid (here in diamond shape) to aminoacylate the RNA bound by the BP.

Due to the entirely probabilistic nature of the “chemical code” only very few of the HDPs will possess this property. We call these HDPs RNA-binding peptides (RBPs) (Figure 2a). As a result of proximity, most RBPs, would likely bind and increase stability of the RNA complexes that produced them. The lack of information transfer from template RNA to RBP would hamper any feedback to select peptides with better stabilizing properties. The absence of selection could be overcome if we consider a particular kind of RBP—one having the property to interact/attract with a specific amino acid or a group of amino acids of similar structure. Such a peptide would, (1) facilitate aminoacylation bringing reactants in proximity and (2) it would necessarily bias the composition of the synthesized peptides (Figure 2b). We call these peptides “bridge peptides” (BP).

As the BP is a short peptide, it would not possess activity specific for longer folded ones. As a result, the function(s) would directly reflect the properties of the incorporated amino acids. One possible mechanism of BP action is to link amino acids with opposite charge. The positively charged end would contact RNA and the negatively charged would attract amino acids of positive charge (their incorporation would provide the greatest RNA binding ability of RBP and greatest stability benefit to the RNA complex). The rest of the residues in the BP would facilitate the requirements like space between the charged amino acids or shape for bridging the RNA and the amino acid with some degree of specificity.

### 2.6. Step 5. “Proto-Translation”

**Figure 3:**
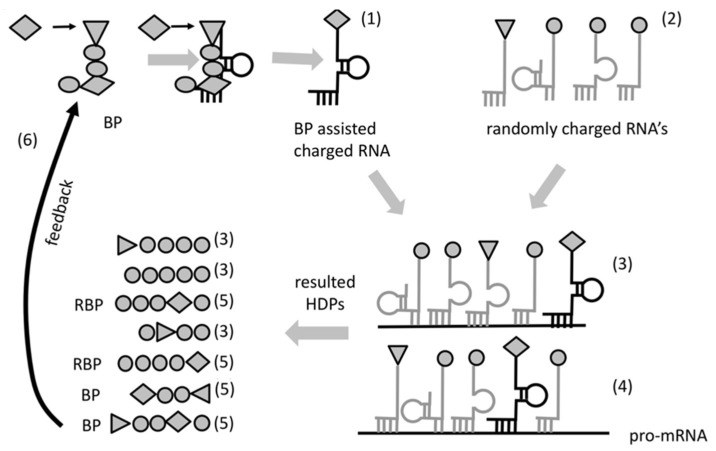
Transition from non-coded to coded peptides. Initially, non-coded BP assembled by chance are involved in specific aminoacylation of certain RNAs (**1**). HDPs are formed (**3**) by both BP-aminoacylated RNAs (bold) (1) and a variety of randomly charged RNAs (light grey) (**2**) in cycles as described in Figure 1 where same RNA may hybridize with many aminoacylated RNAs. The newly formed HDPs are different variants and most are without significant contribution to RNA stability (**3**). In some HDP variants however, RBP or BP are synthesized using BP-charged RNA (**4**). The hybridization process between BP-aminoacylated RNAs and ”template” RNAs (**4**) (pro-mRNA) place a specific amino acid in a specific position relative to the other amino acids in a way that reproduce BP (**5**) or RBP (**5**). This BP is involved in charging another RNA that has a similar sequence (**6**). At this point the cycle is closed increasing the chance that the newly produced BPs would be involved in a new round of BP formation. The feedback cycle (**6**), (**1**), (**4**), and (**5**) provides stability due to the fact that the BP increases the chance to reproduce stabilizing forms of RBP and BP. The information in that case is transferred from the sequence of BP assisted aminoacylation via pro-mRNA to the BP and RBP: A Darwinian selection is established.

Most likely the specificity of interaction between BP and its amino acid might have been very low. Even so, this preference would make the probability of insertion of the cognate amino acid into newly formed hybridization-dependent peptides higher than random. This property (to bind simultaneously a specific RNA and attract specific amino acid or group of amino acids) would enable for the first time the sequence of a template RNA to determinate the sequence of a newly formed peptide (Figure 3). We call this primitive form of coding “protocode”. The key property of the protocode is to define the introduction of the same or similar amino acid into the same place in the sequence of a HDP making possible Darwinian selection for stability. The “bridging” process would be quite inefficient at first and only few (or just one) amino acids would be incorporated due to the protocode. However, the placement of the same amino acids (including rare ones) in the same location relative to the sequence of the template RNA would have, as a consequence, the increase of the portion of functionally active peptides available in the selection pool (Figure 3).

While both BPs (and RBPs) could “work” within different RNA complexes, the ones that matter, in view of selection, would be those that stabilize their native complex. Selection forces would favor BPs that put “useful” amino acids at “successful” positions augmenting the fitness of RBPs. This would result in a positive feedback loop that would increase the chance of synthesis of peptides augmenting the stability of the RNA-peptide complexes. The contribution of BPs towards increased stability of their native complexes would be a consequence of the higher complexity of the synthesized HDPs within these complexes (Figure 3).

### 2.7. Step 6. Replication

At this point though, only non-enzymatic RNA replication would exist with the support of wet/dry natural cycles. Even if BPs would increase the stability of the RBP/BP producing complexes, they would be degraded eventually in the absence of enzymatic replication of RNA. The RNAs of the RNA-peptide complexes would change constantly. The supply of random RNAs (able to recombine with the RNA(s) already present in the RNA-peptide complexes) would be a permanent source of variability providing new properties to the HDPs—importantly to the RBPs and BPs subsets of these. One type of peptide would have a revolutionary effect on stability of RNA-peptide complexes and would be established relatively early—the RNA replication peptide (RRP). The consequence of having RRP would be the reproduction of the BP-stabilized RNA-peptide complexes. RRP most likely would derive from a BP or RBP and would facilitate the formation of phosphodiester bonds at temperatures at which hydrogen bonds could exist. The catalytic center of the nucleotidyltransferases and all modern polymerases is composed of two metal ions (like Mg++) positioned at a specific distance in a way that the first Mg++ deprotonates the 3′-OH of the RNA primer and attacks the alpha-phosphate of the incoming NTP. The second Mg++ stabilizes the NTP moiety [93,94]. We imagine that RRP would have been a peptide capable of “holding” two Mg++ ions (or possibly two Fe++) at the required distance utilizing negatively charged amino acids spaced via neutral amino acids, in a way similar to the modern highly conservative DxD polymerase motif. While experiments to directly model this notion are currently missing, phylogenetic studies [95] showing that the most conservative polymerase domain contains aspartic residues which coordinate catalytic magnesium ions, suggesting that RRP could have been selected soon after Darwinian selection was established.

### 2.8. Step 7. the Evolution of Functions

We presume that following this initial stage all components of the translation system would co-evolve in a stepwise way. For example, from certain point on, RNAs being aminoacylated and involved into peptidyl transfer should specialize to serve as tRNAs only. That would be possible if the longer peptides, evolved forms of BP, could recognize a specific 3D structure of the proto-tRNAs. In the predominantly GC sequences found in the RNA-peptide complexes, CCA-3′ is a particularly good recognition pattern. From that moment on CCA-3′ would have likely become the signature of the aminoacylation site of all tRNAs.

Specialization of ribosomal Large Subunit—LSU will start with evolution of peptidyl transferase center (PTC). This event by itself would have been enormously beneficial for stability, since the peptidyl transfer had so far occurred “by chance”—due to close proximity of two adjacent amino acids brought together by hybridized aminoacylated RNAs.

**Figure 4:**
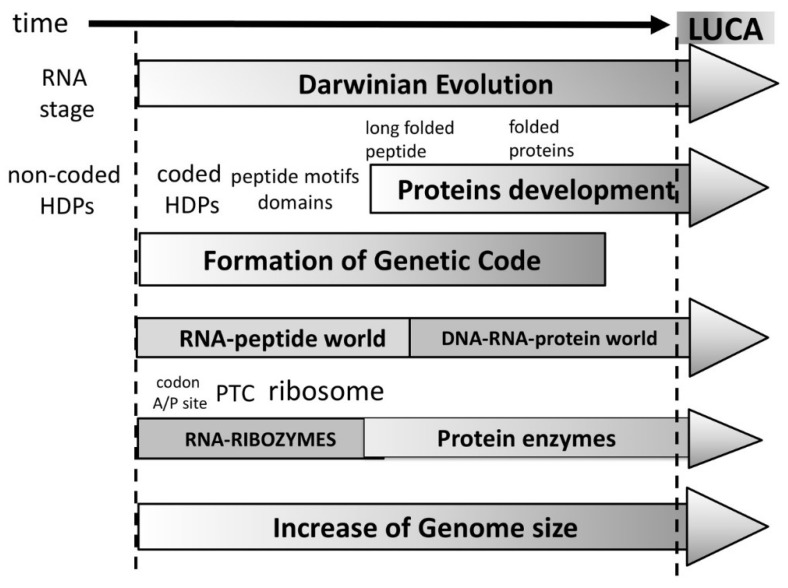
Outline of most significant events leading to the Last Universal Common Ancestor (LUCA). The events that occurred during the same time period are shown vertically. Transition from non-coded to coded peptides gave rise to Darwinian evolution as described in Figure 3 and continue ever since. Proteins develop from small sized peptides with only a fraction of coded amino acids to formation of motifs and domains with increased coded content. At a later stage the peptides became able to fold and form catalytic centers leading to formation of proteins with fixed 1st and last amino acids. At the same time standard genetic code expands to cover more codons for at least 20 amino acids. By the time when the first proteins are fully formed the genetic code is established as we know it. During the period of short peptides and lack of folded peptides/proteins (RNA-peptide world) certain RNAs with catalytic functions were selected (ribozymes) to perform critical catalytic functions. This is time when the ribosome obtained its basic shape. By the time the peptides begun to fold and acquire new enzymatic functions, the ribozymes were already established and could not be replaced, but started to co-exist together with proteins. The genome size increased proportionally covering the information for all proteins and ribozymes. The Last Universal Common Ancestor (LUCA) emerged when all of its components—the ribozymes, genetic code, and proteins were functioning in full synchrony.

The evolution of peptides to proteins would occur from small motif to domains and finally—folded proteins. The functional repertoire would evolve as well from the very limited functional ability of short peptides to longer folded peptides and finally to proteins forming complex catalytic centers with almost unlimited possible functions. Therefore, the peptide/protein evolution offers a reasonable explanation why the ribozymes have been selected in the first place. The ribozymes, including the ribosome would have been selected during the RNA-peptide world where sophisticated folded proteins were still non-existent (Figure 4). An important point that should be made regarding the evolution of functions is that with the increase of complexity the main driving force of the evolutionary process is no longer simply stability, but the competition of separate self-sustained entities. These would presumably need to have some form of separation from the environment (i.e., in the form of lipid vesicles) and should necessarily contain stabilized peptide-RNA complex(es) to perpetuate their information and functional identities and serve as the raw material for Darwinian selection.

### 2.9. Step 8. the Emergence of Standard Genetic Code (SGC)

According to our hypothesis, the BP would influence the formation of the first proto-codon usage in the proto-translation system. Simultaneously, with the process of adding new codes, the role played by amino acid abundance (the “chemical code”) would decrease. The increase in complexity and the takeover of coded incorporation for all 20 canonical and some non-canonical amino acids should have been a gradual process of co-evolution of tRNAs, proto-forms of aaRS, the ribosome and peptide functions. The standard 3 base codon as we know it should be established as a result of the most advantageous fit of tRNA/ribosome structure with the functional requirement of amino acids.

Each step of addition of new tRNA/amino acid combination should have been tolerable in its intermediate form—for example, lysine could have started to aminoacylate formerly arginine-specific tRNA, since both have similar charge and structure and initially may have covered similar functions. We imagine the same could have happened with tRNAs having different anti-codons but encoding the same amino acid. This would lead to the same amino acid being positioned more frequently in different peptide positions. These tolerable intermediate changes would have been possible due to the relative simplicity of the early complexes. With the acquisition of catalytic functions by the polypeptides, changes to the coding system would become more difficult and eventually impossible. However, in the meantime, the co-evolution of the protein coding apparatus would have led to the efficient translation system of LUCA with “well-fitted” tRNAs, aaRS, ribosomes and almost 600 genes working in synchrony. A similar mathematical concept has already been developed by Reijer Lenstra through codon progressive symmetry breaking [96].

## 3. Discussion

We have developed an RNA-peptide hypothesis for the origin of life with the principal assumption that if a structure-function is beneficial and selected during the origin of life, it could not be replaced later. This concept, called “the rule of continuity”, has been suggested before [43,52,97], but its implications have not been sufficiently explored. Clearly, the earlier a given structure-function had emerged the more processes developed later would depend on it and its replacement by a (more efficient) variant would incur a higher fitness cost. While major changes had taken place during evolution (i.e., replacement of genes, functions and organs)—all known such examples had taken place by the selection of a pre-existing “backup feature” turned more suitable for the new environment.

The application of the rule of continuity to translation, suggests that aaRSs (or their proto-forms) should have been important from the very inception of the process. Yet, phylogenetic analyses of modern aaRSs bring up the “protein evolution paradox”, highlighting the fact that by the time the ancestors of the aaRSs gave rise to specific proteins, extensive evolution of their domains had already produced a substantial diversity of other enzymatic and nucleotide-binding folds. This was argued to require high-fidelity translation and imply that specificity of amino acid–codon correspondence was initially determined by RNA molecules [45], followed later by a 

“…takeover by aaRSs of the key role in the pairing of amino acids with the cognate anticodons [9]”.

The transition of the chemical nature of the molecules determining the genetic code constitutes a major breach of the rule of continuity. Our hypothesis avoids this by postulating the existence of a proto-form of aaRS (bridge peptide and its evolved forms) involved in codon formation by facilitating proximity between a specific RNA and specific amino acid. In addition, BP is capable to stabilize the RNA-BP complex forming a feedback loop (Figure 3) where the most stable form is also the coded one. Consequently, BP would enable Darwinian selection, which in turn would further improve the specificity of RNA aminoacylation of this primitive translation system.

We explain the beginning of translation as a process of mutual cooperation between RNA and peptides as suggested by Williams and colleagues [5,28,52], which continued by evolutionary increases in complexity and specialization resulting in modern tRNAs, rRNA, and ribosomal proteins. Thus, our hypothesis explicitly does not need the “final” variants of aaRS to be involved in the setup of the genetic code, explaining the apparent discrepancy between the rule of continuity and the phylogenetic data [54,55].

The rule of continuity is also at odds with the lack of any remnants of “RNA world” replication machinery, preserved by evolution, although ribozymes capable of RNA replication have been selected in the lab [14,15,16,17,18,19,20]. At the same time an RNA structure carrying out a similarly crucial function—translation—has been preserved, arguing for the validity of the rule of continuity. Even “compromise” models of RNA replication such as the triplet model [7,24] suffer the same deficiency breaking the rule of continuity, and as of today, any template-directed replication is performed by proteins adding nucleotides one-by-one. This strongly argues that a ribozyme capable of RNA replicating activity never existed in the first place and this function began being performed by a peptide RNA polymerase. Alternatively, ”hybrid” RNA-peptide could have supported RNA replication. Experimentally, peptides that increase the ribozyme ligase activity by as much as 18,000 fold have been selected [90], highlighting the importance of the peptide component in the process. In our hypothesis, the lack of an RNA-based RNA replicase could be filled by chemical polymerization [75,98], which would preferentially amplify more stable, “longer-living” RNA molecules. Such a low fidelity process could be adequate for the functionally poor RNA species that existed at that time. On the other hand, a peptide able to catalyze RNA polymerization could be very simple— just capable of holding RNA and Mg^++^ or Fe^++^ in appropriate spatial proximity. Such an RNA replication peptide would provide a huge leap in stability, as part of an already established RNA-coded peptide complex. In addition, it seems likely that an efficient RNA replication ribozyme during early evolution would diminish RNA reactivity by forming complementary chains whose hybridization would eliminate any significant 3D structures. While, ways to form single stranded RNA could be envisaged—i.e., by toehold-based strand displacement [99], it is unlikely that these would be sufficient to counter the decrease of unhybridized sequences and 3D structure brought about by an efficient RNA replication ribozyme. Without a complex 3D structure provided by limited complementarity, hybridization of aminoacylated RNAs and synthesis of HDPs would be unlikely to occur. Therefore, our model views a replication ribozyme during this initial phase of evolution as counter-productive to the maintenance of a stable RNA-peptide complex.

Penny and coauthors [53] have formulated three major advantages of RNA-centric models for origin of life due to heredity.

“1. Without heritable templates, it is unlikely that multiple copies of a favorable peptide can be produced accurately and repeatedly.

2. Peptides produced in a pre-genetic phase cannot improve for lack of preferential replication of favored forms, while genetically encoded RNA enzymes would predominate being improved via Darwinian evolution. 

3. Complexity of modern translation is the result of an optimization process, impossible without natural selection of the early ribosomal machinery, on the code itself, and on early genes.”

In our hypothesis, heredity begun because of BP and will exists as a result of increasing stability of complexes. The ability of the bridge peptides to bring specific amino acids into the same position would improve the odds to reproduce similar HDPs and selection would lead to more copies of the more favorable ones. The accuracy of bridging would also improve by selection (Figure 3) allowing for increases in fitness and complexity. Thus, stability selection of small partially coded peptides obviates the initial need for high fidelity peptide synthesis. It would develop with the improvements of bridging process turned translation, being itself (as suggested by Poole and Penny) a result of optimization.

### 3.1. “RNA World” or “RNA-Peptide” World?

Several hypotheses have been put forward that explore the advantages of RNA-peptide cooperation during the initial stages of the origin of life. Regarding the difficult-to-explain RNA replicating ribozyme George Fox in his “abbreviated RNA world” hypothesis suggested: 

“If peptide synthesis arises quickly, then there will neither be time nor need for extensive catalysis of biochemical reactions by RNA. If reasonable, the rapid appearance of a translation system may even eliminate the need to validate the RNA world by demonstrating the self-replicating RNA system that has proven experimentally difficult to achieve. [46]”

Other authors, mathematically modeled the likelihood of emergence of a self-assembled coding system based on one heteropolymer (RNA Coding World) or two different heteropolymers (Protein Coding World), concluding the second is far more plausible than the “RNA world” scenario [50].

While formation of the non-coded HDPs in our hypothesis could be considered a ribozyme function, the lack of positive feedback for selection means that it is not carried out by a self-sustained entity. Similar to Williams and team [5,28], our hypothesis assumes that life originated after the cooperation between RNA and peptides had started or to be more precise: At the moment when the sequence of RNA began to determinate the sequence of peptide, the life began.

Thus, it replaces an RNA-only self-sustained stage (RNA-world), with an RNA stage capable to produce HDPs (Figure 4). A very strong argument in favor of the “RNA world” is the existence of natural ribozymes [21]. In our hypothesis ribozymes are relics from the RNA-peptide world not from RNA world. During the period of time between the first coded amino acid until the formation of folded long peptides, functional diversity of the existing peptides would have been insufficient and ribozymes would have been selected for certain functions. As a consequence of the rule of continuity some would be retained [21]. A clear illustration of that is the fact that the most important ribozyme is the ribosomal PTC working without nearby proteins, since there were no proteins at the time it was functionally selected. As the rule of continuity dictates, the PTC could not be replaced by protein-based peptidyl-transfer enzymes, because there were no long proteins to do so. Another example includes the RNA part of RNase P (M1 RNA)—a molecular fossil which most likely was involved in tRNA maturation [12,100,101]. We assume that M1 RNA evolved in a period when tRNA was also evolving. tRNA evolution improved its peptidyl transfer function with the ribosome, therefore it is unlikely that M1 RNA have evolved in a non-coded peptide free environment.

This applies for ribonucleotides (Nicotinamide adenine dinucleotide (NAD) and flavin adenine dinucleotide (FAD) capable of electron exchange for oxidation-reduction reactions. Theoretically, these reactions could be carried out by folded proteins, however these were not available and the selection went in that direction.

Williams and Petrov developed a concept [28,102] in which they advocate PTC as being the very first structure to determinate the origin of life. In contrast, our hypothesis does not hold this statement, despite the fact that PTC would have had a dramatic impact on peptidyl transfer efficiency and consequently on survival. Translation in our model started from an RNA complex, which will correspond to the future codon recognition center in SSU, and tRNA interacted with mRNA. Such a view is supported by phylogenetic analyses by Caetano–Anolles and team showing that regions around A-P sites (helix 44) of the 16S evolved earlier than the PTC [103]. Currently, PTC first hypothesis is a subject of intensive debate [102,104]. As an additional clue which may help solving this dispute, we could observe the process of assembly of the modern ribosome just before the start of protein synthesis. In that process, the LSU joins after the formation in pre-initiation complex containing the SSU, mRNA, the first tRNA-Met (reviewed by [105]).

We assume that immediately after the initial stabilization of the RNA/RBP/BP complex, co-evolution of the codon/A/P sites, PTC, tRNA, and the first peptides involved in RNA replication, ribosome stabilization, and more will occur (Figure 4). Therefore, there should be motives in rRNA (16S and 23S) and tRNA left over from ribosomal proteins, polymerases, and other genes important during this early stage of development. In line with this expectation, recent analyses indicated that the ribosomal RNA is not just a structural scaffold, but also a vestigial remnant of a primordial genome. The *E. coli* 16S and 23S RNA contain the entire set of transfer RNAs for all twenty amino acids as well as sequences encoding key fragments of ribosomal proteins, polymerases, ligases, etc. [106]. According to the authors, these findings argue in favor of a ribosomal self-replicating stage. In light of our hypothesis, these data support both an RNA-peptide world and the co-evolutionary development of protein synthesis apparatus.

Within this framework we could envision the evolutionary fate of aaRSs, based on the urzyme hypothesis [29,30]. The “urzymes” are ~120 residue fragments of the two aaRS superfamilies, capable of interacting with tRNA acceptor stem. Urzymes retain significant portions of catalytic proficiency and specificities of full aaRSs. Urzymes possess a conserved 46-amino acid “protozyme” motifs (itself formed by two inverted copies of a 23-mer fold) in the ATPase binding sites of both classes of aaRSs [29,30,50,58,59], suggesting that urzyme-like structures may have served as proto-aaRS. The urzyme model assumes the key role of urzymes as aaRSs precursors during code formation. The authors postulate the beginning of the protozyme as a process of stability generated by complementary van der Waals forces between opposite chiralities of amino acids and ribose of RNA. The next step, the specific interactions and development of the code is attributed to stereochemical interactions between amino acids and RNA [29,30,50,58,59]. As we discussed before, the stereochemical way of codon formation contradicts the rule of continuity, but the BP we hypothesize can explain the gap between non-coded peptides and the emergence of the protozyme. The protozyme suggested from Carter and Wills however does not interact with tRNA, therefore it is difficult to “link” our concept for BP with the protozyme as it is described. Almost certainly, an evolutionary path from BP to protozymes to urzymes to aaRS that would fully satisfy the rule of continuity would require a modified protozyme that is capable of proto-tRNA interaction.

Caetano-Anolles and Caetano-Anolles published their variant of the origin of translation by including peptides named elementary functional loops (EFL) [49]. EFLs were synthesized by statistically biased condensations and interacted with RNA hairpins by stereochemical forces. According to the authors, the development of EFL would lead to proto-domain(s), fold family (FF) and fold super family (FSF) and were the basis of urzymes and all proteins [49]. Any initial complex however in order to begin its evolution would need a positive feedback “information-structure”, therefore the link between RNA and amino acids involved in the formation of new peptides must be established first. The step from EFL to proto-domain as Caetano–Anolles and Caetano–Anolles suggested is a substantial leap of stabilization and would increase the size of the EFL, which is unlikely to occur by chance, lacking the positive feedback as we suggest. The BP according to us is simple enough to emerge by accident and provides the necessary positive feedback due to hybridization events, which logically may lead to the FF and FSF’s from the Caetano–Anolles and Caetano-Anolles concept. 

### 3.2. Formation of Standard Genetic Code

In their latest review Koonin and Novozhilov described the biggest challenges for any theory explaining the origin of the Standard genetic code (SGC) and translation: 

“Evolution of the code is intimately linked to the origin and evolution of the translation apparatus itself... Evolution of translation involves an intrinsic and formidable paradox: A high-fidelity translation system requires a number of functional proteins, but the maintenance of such proteins is impossible without a reliable translation system.” [45]. Essentially the same argument (regarding the accuracy of the translation system required for protein evolution and the need to evolve one) has been raised before [9]. This is the major reason for RNA world models to view the formation of the genetic code and that of translation as two separate processes, the former coming from an RNA world with no need for proteins and the latter as a result of polymer transition [107,108]. 

Our model is completely opposed to such a concept. We view code and translation as impossible to separate. This notion is usually countered by the argument that evolution had no foresight to create life. However, we believe foresight is unnecessary. The sole driving force of evolution from HDPs to coded translational system in our hypothesis is the selection of more stable forms. The one capable of coded translation would also be the most stable. Stability selection is the reason why the amino acids being incorporated by bridging will be those that contribute most to the overall stability of the complex, not the most abundant. Precisely because the nature has no foresight, it is unlikely that the relative abundance of the amino acids during the prebiotic synthesis would fit well to the “needs” of newly formed RNA-peptide complexes. For that reason, contrary to Higgs [88], in our view amino acids cannot be classified as early/late based on abundance.

Our hypothesis also avoids the apparent paradox of the need of a high fidelity translation system for protein evolution before it had evolved by postulating the gradual interrelated increases in complexity and fidelity of the translation apparatus composed of co-evolving components. It thus suggests a path for the Darwinian evolution of a self-sustained RNA-peptide entity.

In summary, our hypothesis of the origin of life differentiates the following initial three major periods in its timeline (Figure 4):

1. Prebiotic period during which very short 4–20 nt of the RNA and activated amino acids were synthesized.

2. “RNA stage” in which RNA capable of recombination and importantly performing a low efficiency peptidyl transfers resulted in non-coded short hybridization-dependent peptides.

3. “RNA-peptide world” beginning when the first amino acid was incorporated by bridging (Figure 3). This initiated Darwinian selection, which in turn led to longer and functional peptides as the number of coded amino acids grew. In that period, the genetic code and translation apparatus began to developed simultaneously. Functional limitations of early peptides allowed the selection of ribozymes in that period. Ribozymes carried out many important functions. The first metabolic molecules with ribonucleotide nature like NAD, FAD, Coenzyme A, etc., were selected in proto-metabolic reactions. The ribosome started to take shape, beginning from the A/P sites (h44) via co-evolution with the PTC center, tRNA, and aaRS. Domains diversified and the first true proteins with specific start and end formed, marking the end of the RNA-peptide world.
